# Drug Combination Studies of the Dipeptide Nitrile CD24 with Curcumin: A New Strategy to Synergistically Inhibit Rhodesain of *Trypanosoma brucei rhodesiense*

**DOI:** 10.3390/ijms232214470

**Published:** 2022-11-21

**Authors:** Carla Di Chio, Santo Previti, Fabiola De Luca, Marta Bogacz, Collin Zimmer, Annika Wagner, Tanja Schirmeister, Maria Zappalà, Roberta Ettari

**Affiliations:** 1 Department of Chemical, Biological, Pharmaceutical, and Environmental Sciences, University of Messina, Viale Ferdinando Stagno d’Alcontres 31, 98166 Messina, Italy; 2 Institute of Organic Chemistry & Macromolecular Chemistry, Friedrich-Schiller University of Jena, Humboldtstraße 10, 07743 Jena, Germany; 3 Institute of Pharmaceutical and Biomedical Sciences, University of Mainz, Staudingerweg 5, 55128 Mainz, Germany

**Keywords:** cysteine protease, rhodesain inhibitors, dipeptide nitrile, curcumin, combination studies

## Abstract

Rhodesain is a cysteine protease that is crucial for the life cycle of *Trypanosoma brucei rhodesiense*, a parasite causing the lethal form of Human African Trypanosomiasis. **CD24** is a recently developed synthetic inhibitor of rhodesain, characterized by a nanomolar affinity towards the trypanosomal protease (*K*_i_ = 16 nM), and acting as a competitive inhibitor. In the present work, we carried out a combination study of **CD24** with curcumin, the multitarget nutraceutical obtained from *Curcuma longa* L., which we demonstrated to inhibit rhodesain in a non-competitive manner. By applying the Chou and Talalay method, we obtained an initial additive effect at IC_50_ (f_a_ = 0.5, Combination Index = 1), while for the most relevant f_a_ values, ranging from 0.6 to 1, i.e., from 60% to 100% of rhodesain inhibition, we obtained a combination index < 1, thus suggesting that an increasingly synergistic action occurred for the combination of the synthetic inhibitor **CD24** and curcumin. Furthermore, the combination of the two inhibitors showed an antitrypanosomal activity better than that of **CD24** alone (EC_50_ = 4.85 µM and 10.1 µM for the combination and **CD24**, respectively), thus suggesting the use of the two inhibitors in combination is desirable.

## 1. Introduction

Human African Trypanosomiasis (HAT), also known as sleeping sickness, is a parasitic disease widespread in sub-Saharan Africa, where it represents a relevant cause of death [1]. HAT is induced by two subspecies of Trypanosoma: *T. brucei gambiense*, which is able to cause the chronic form of HAT and widespread in western and central Africa, and *T. b. rhodesiense*, which is common in eastern and southern Africa, and responsible for the rapid-onset high death rate HAT [2].

Current HAT therapy is based on a few dated drugs with a limited spectrum of action, toxicity, and problems related to the parenteral route of administration. At present, the first-line treatment of the *gambiense* HAT is based on nifurtimox–eflornithine combination therapy (NECT), in which nifurtimox is used off-label, since it was approved for Chagas disease [3]. Recently, a new orally administered molecule, i.e., fexinidazole, was introduced in therapy, with an improvement of patient compliance; however, its approval is limited to the gambiense form of HAT [4,5]. From this perspective, there is an urgent need to identify new targets to develop new drugs that are also active on the lethal rhodesiense form of HAT. 

Several strategies have been put in place for the development of novel antitrypanosomal agents [6,7,8,9]. In this scenario, rhodesain, the main cysteine protease of *T. b. rhodesiense*, is an attractive target, since it mediates essential processes for parasite survival and disease progression; thus, it has become one of the main targets for the drug discovery process of new antitrypanosomal agents [10,11].

Rhodesain owes its importance to its various functions: (a) it is responsible for the crossing of the blood–brain barrier of the human host [12], thus inducing the neurological stage of HAT; (b) it is involved in the elusion of the host immune system, since it takes part in the turnover of variant surface glycoproteins of the trypanosome coat and degrades the host immunoglobulins [13,14]; (c) last, it shows a significant proteolytic activity in lysosomes, since it is involved in the degradation of intracellularly transported host proteins, as well as in parasite proteins. For these reasons rhodesain is considered an important target for HAT treatment [10,11].

In this area of medicinal chemistry, and over the last few decades, our research group has been actively involved in the development of novel rhodesain inhibitors [15,16,17,18,19,20,21,22,23,24,25].

More recently, starting from consistent literature data that demonstrated the ability of the nitrile function to react with the catalytic cysteine of rhodesain and also of several cathepsins [26,27,28], we have developed a new class of dipeptide nitriles, as potent rhodesain inhibitors [29].

**Figure 1:**
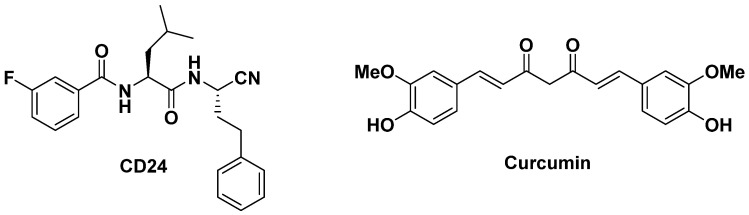
Structures of **CD24** and curcumin.

Within the most interesting compounds, the dipeptide nitrile **CD24** (Figure 1) showed the highest binding affinity towards rhodesain (*K*_i_ = 16 nM), coupled with a good antiparasitic activity, i.e., EC_50_ = 10.1 ± 0.5 µM. We also proved that **CD24** directly binds to the active site of rhodesain, acting as competitive inhibitor [29].

Considering our expertise in drug-combinations [30,31,32], we carried out a combination study of the novel identified lead compound **CD24** with curcumin (Figure 1), a multitarget nutraceutical obtained from *Curcuma longa* L., which we demonstrated to inhibit rhodesain in a non-competitive manner [31].

Our aim was to evaluate, according to the Chou and Talalay method [33,34], if additive or synergistic effects occur in rhodesain inhibition, when we combine the synthetic inhibitor **CD24** and the nutraceutical curcumin, considering that there can be many advantages of drug combinations, e.g., reduced risk of toxicity for the human host by reduction of individual dose or the use of lower amounts of poorly soluble compounds [35].

## 2. Results and Discussion

**CD24** and curcumin were tested against recombinant rhodesain by using Cbz-Phe-Arg-AMC as a fluorogenic substrate [36]. We initially carried out a screening at 100 µM, 1 µM, and 0.1 µM, to evaluate the range of activity of the two inhibitors. **CD24** and curcumin were then separately tested in two independent experiments, each performed in duplicate. Seven different concentrations were selected for **CD24** and curcumin, starting from the minimum dose required to inhibit the enzyme, to that necessary to fully suppress the rhodesain activity. In more detail, we used concentrations in the range 0.05–20 µM and 5–100 µM for **CD24** and curcumin, respectively.

**Figure 2:**
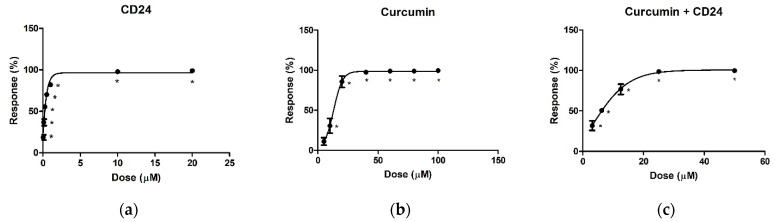
Dose–response curves for rhodesain inhibition by **CD24** (**a**), curcumin (**b**), and **CD24** + curcumin in combination (**c**). Each experiment was performed two times, each in duplicate with * *p* < 0.0001 vs. no inhibitor.

IC_50_ values were calculated from dose response–curves, as shown in Figure 2: 0.2 ± 0.01 µM for **CD24** and 12.3 ± 0.9 µM for curcumin.

In a subsequent experiment, five data points were established for both compounds (1/4 × IC_50_, 1/2 × IC_50_, IC_50_, 2 × IC_50_, and 4 × IC_50_, Table 1), with the aim of evaluating if a synergistic, additive, or antagonist effect occurred in the combination study of the inhibitors. In this assay, the combination of **CD24** and curcumin (molar ratio 1:62) provided an IC_50_ value of 5.6 ± 0.4 µM. 

**Figure 3:**
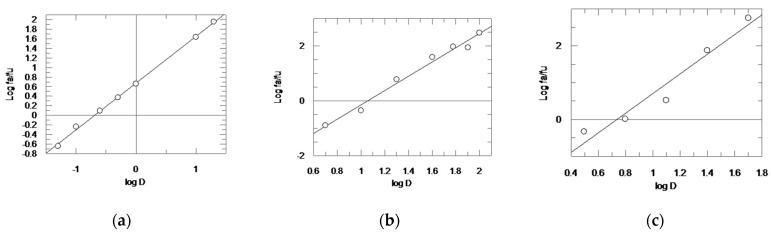
Median effect plot for **CD24** (**a**), curcumin (**b**), and **CD24** + curcumin in combination (molar ratio 1:62) (**c**). D is the dose, and f_a_ and f_u_ are the affected and the unaffected fraction of rhodesain activity, respectively, by dose D.

We then converted each dose–response curve into a median effect plot, which was obtained by plotting on the *y*-axis the log (f_a_/f_u_) versus the log (D) on the *x*-axis (Figure 3). In the median effect plot the maximum response corresponds to 1, instead of the 100 of the dose–response curve. Therefore f_a_ + f_u_ = 1, where f_a_ corresponds to the “affected fraction”, i.e., the percentage of enzyme that has been inhibited, while f_u_ is the unaffected fraction, i.e., the residual enzyme activity. The slope of the straight line of each median effect plot is the “m value”; in detail, **CD24** showed m_1_ = 0.9757 and curcumin m_2_ = 2.6066, while for the combination assay, we found m_1,2_ = 2.6705, with a molar ratio **CD24**/curcumin of 1:62.

Once we had calculated the three different m values using Grafit sotware (Version 5.0.1.3; Erithacus Software Limited, East Grinstead, West Sussex, UK), we established the doses which were able to induce each percentage of rhodesain inhibition by means of the median effect equation D = IC_50_ [f_a_/f_u_]^1/m^ [33,34].

Starting from the assessment that **CD24** is a competitive inhibitor of rhodesain [29], while curcumin acts as non-competitive rhodesain inhibitor [31], as previously demonstrated by our research group, and with the aim of determining the inhibitory effect given by the combination of **CD24** and curcumin, we used the Chou–Talalay method to evaluate the multiple drug effects [33,34].

In more detail, we calculated the combination index (*CI*), which expresses the nature of the inhibition towards the target enzyme when two drugs are tested in combination.

In particular, it is well known that a *CI* > 1, *CI* = 1, and *CI* < 1 generally correspond to an antagonistic, additive, and synergistic effect, respectively [33,34]. The *CI* for mutually non-exclusive drugs, which act independently, was calculated as follows:

*CI =* [(*D*)_1_*/(IC*_50_)_1_] *+* [(*D*)_2_/(*IC*_50_)_2_] *+* [(*D*)_1_(*D*)_2_]/[(*IC*_50_)_1_(*IC*_50_)_2_

where (*IC*_50_)_1_ and (*IC*_50_)_2_ were already obtained using dose–response curves, while the *D*_1_ and *D*_2_, able to induce a specific percentage of rhodesain inhibition were obtained using a median effect equation. 

**Figure 4:**
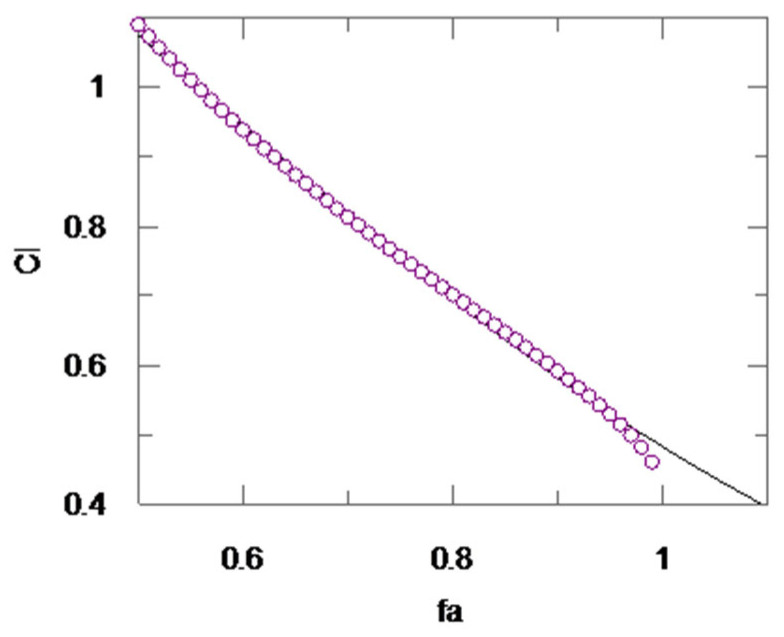
Computer-generated graphical presentation of the combination index (*CI*) vs. the fraction affected (f_a_), i.e., the effect of reduction of rhodesain activity exerted by a mixture of **CD24**–curcumin (molar ratio 1:62).

Grafit software was used to determine the *CI,* ranging from 50% to 100%, of rhodesain inhibition (Figure 4). Starting from the IC_50_, which is normally taken to determine the activity of a novel inhibitor, we observed an initial additive effect, since *CI* resulted = 1, according to Chou’s rules [33,34,37].

Interestingly, for the most significant f_a_ values, which ranged from 0.6 to 1 (i.e., from 60% to 100% of rhodesain inhibition), an increasing synergistic effect was detected when **CD24** and curcumin were used in combination (Table 2).

**Figure 5:**
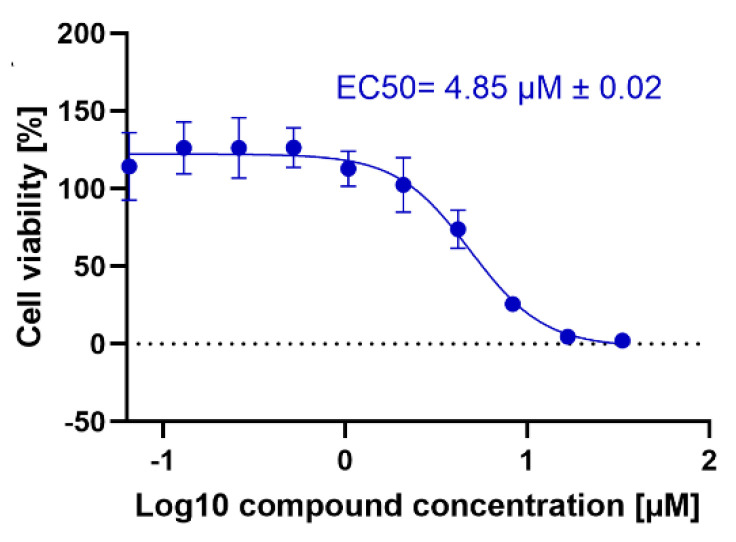
Dose–response curves of the combination **CD24** + curcumin against *T. b. Brucei*.

Considering our previously recorded activity of **CD24** [29] and curcumin [31] alone against *T. brucei brucei* (Table 3), we decided to test the two inhibitors in combination (molar ratio 1:1) by obtaining EC_50_ = 4.85 ± 0.02 µM (Figure 5). Overall, the obtained data led us to assume that the use of our synthetic inhibitor **CD24** in combination with curcumin led to an improvement of its antitrypanosomal activity (EC_50s_ = 10.1 ± 0.5 µM [29] vs. 4.85 ± 0.02 µM), thus suggesting a fruitful use of the drugs in combination. 

**Figure 6:**
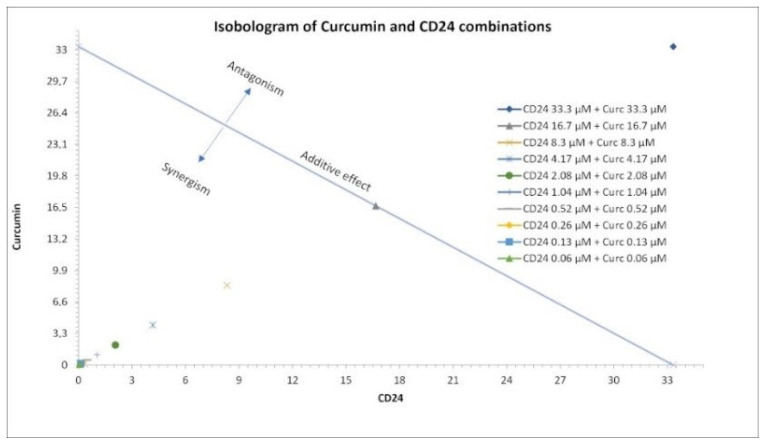
Classic isobologram for CD24 and curcumin with used doses on the *x*- and *y*-axis.

A classic isobologram analysis was performed to evaluate which of the used doses of **CD24** and curcumin, used in a molar ratio 1:1, was able to produce a synergistic effect when combined against *T. b. brucei* (Figure 6). If the combination data points fell on the hypothenuse (i.e., the dose of 16.66 µM), an additive effect was indicated. If the combination data points fell on the lower left (e.g., all the doses ranging from 0.06 µM to 8.33 µM) a synergism was indicated. While for the sole combination point that fell on the upper right (i.e., 33.33 µM), an antagonistic effect was indicated.

Finally, the cytotoxicity of **CD24,** curcumin alone, and **CD24** in combination with curcumin was assessed towards HEK293 cell lines, by using the range of concentrations 70–0.5 µM. In both cases, no cytotoxic effects were observed up to 70 µM. 

All in all, the nutraceutical showed the highest selectivity index (SI), while the synthetic inhibitor alone showed the lowest SI. The combination of **CD24** + curcumin showed a SI slightly lower than that of curcumin, thus signifying a productive use of the inhibitors in combination, considering their strong synergistic action against rhodesain. 

## 3. Materials and Methods

### 3.1. Rhodesain Inhibition Assays

**CD24** was synthesized as previously reported by our group [29]. Curcumin was purchased from Sigma Aldrich.-Merck Life Science (Milan, Italy) Rhodesain was recombinantly expressed by our group, as previously described by Caffrey et al. [39]. Preliminary screening with rhodesain was performed with inhibitor concentrations of 100 µM, 1 µM, and 0.1 µM, to identify the range of activity of **CD24** and curcumin. An equivalent amount of DMSO was used as negative control. Product release from substrate hydrolysis (Cbz-Phe-Arg-AMC, 10 µM) was determined continuously over a period of 10 min at room temperature. The assay buffer contained 50 mM sodium acetate, pH = 5.5, 5 mM EDTA, 200 mM NaCl, and 0.005% Brij 35, to avoid aggregation and false-positive results. Enzyme buffer contained 5 mM DTT rather than Brij 35. Inhibitor solutions were prepared from stocks in DMSO. As a first step, **CD24** and curcumin were separately tested two times in duplicate in 96 well plates in a total volume of 200 µL. In more detail, we used 0.05 µM, 0.1 µM, 0.25 µM, 0.5 µM, 1 µM, 10 µM, and 20 µM for **CD24**, while 5 µM, 10 µM, 20 µM, 40 µM, 60 µM, 80 µM, and 100 µM were used for curcumin. 

Fluorescence of the product AMC of the substrate hydrolyses was measured using an Infinite 200 PRO microplate reader (Tecan, Männedorf, Switzerland) at room temperature, with a 380 nm excitation filter and a 460 nm emission filter. Results are expressed as IC_50_ values ± SD and were calculated by fitting the progress curves to the 4 parameter IC_50_ equation using GRAFIT software 5.0 (GraFit, version 5.0.1.3; Erithacus Software Ltd.: London, UK, 2006):

$$y=\frac{y_{\max}-y_{\min}}{I+{\left(\frac{\left[I\right]}{{\mathrm{IC}}_{50}}\right)}^{s}}+y_{\min}$$

with y [ΔF/min] as the substrate hydrolysis rate, y_max_ as the maximum value of the dose−response curve, measured at an inhibitor concentration of [I] = 0 μM, y_min_ as the minimum value, obtained at high inhibitor concentrations, and s as the Hill coefficient.

As a second step, **CD24** and curcumin were tested in combination using 5 data points: 0.25 × IC_50F1+F2_, 0.50 × IC_50F1+F2_, IC_50F1+F2,_ 2 × IC_50F1+F2_, 4 × IC_50F+F2_, where F1 = CD24 and F2 = curcumin.

### 3.2. Antitrypanosomal Activity Assay

The parasites used in this study were culture-adapted *T. b. brucei* 449, descendants of the Lister strain 427 [40]. Cytotoxic activity of the combination of **CD24** with curcumin in 1:1 molar ratio against *T. b. brucei* was determined using the ATPlite assay, as described previously [29,36,41,42]. Stock solution of the **CD24** and curcumin in DMSO was prepared by mixing the compounds in a 1:1 molar ratio, for a final concentration of 10 mM. This stock was then used to perform serial dilutions in culture media. The final concentrations applied on the cells were 33.33 µM, 16.66 µM, 8.33 µM, 4.16 µM, 2.08 µM, 1.04 µM, 0.52 µM, 0.26 µM, 0.13 µM, and 0.06 µM. 

### 3.3. Cytotoxicity Evaluation

HEK293 cells were cultured in high glucose DMEM medium with L-glutamine, supplemented with 10% FCS, 20 U/mL penicillin, and 20 µg/mL streptomycin, at 37 °C and 5% CO_2_. Cytotoxic activities of **CD24** and **CD24**/curcumin combination (in 1:1 molar ratio) were assayed using resazurin staining, as described previously [43]. Briefly, prepared compound or compound mix stocks in DMSO were subjected to seven consecutive 1:2 dilution steps in DMSO. The resulting eight dilutions of the compound, ranging from 7 mM to 0.055 mM, were further diluted 1:100 with addition to the wells of poly-lysine-coated 48 well plates containing cells that had been seeded at 60,000 cells/well and incubated for 24 h at 37 °C. The cells were incubated with the compounds for 21 h at 37 °C, after which the culture medium in each well was exchanged for medium supplemented with resazurin (15 µg/mL). After 3 h at 37 °C incubation with the resazurin-containing medium, an aliquot of 100 µL was removed from each well and transferred into a black, clear-bottom 96 well plate, and the fluorescence was measured (excitation: 540–14, emission: 590–20) using a CLARIOstar Plus plate reader (BMG Labtech, Ortenberg, Germany). Cells treated with DMSO alone were used as control. The assay was performed in duplicate.

### 3.4. Statistical Analyses

The statistical analysis of the data was performed using the one-way test (ANOVA) with Dunnett’s multiple comparison test, considering significant differences of *p* < 0.05 with respect to the percentage of rhodesain inhibition of curcumin, CD24, and curcumin + CD24. The analyses were performed with GraphPAD Prism 6 (GraphPad software Inc., San Diego, California). Results are expressed as the arithmetic mean ± standard deviation (SD).

## 4. Conclusions

In summary, in this study, starting from the single activities of **CD24** and curcumin against rhodesain, we investigated the activity of their combination, concluding that at IC_50_ an initial additive effect was observed (*CI* = 1), while for the most significant f_a_ values, i.e., those ranging from 0.6 to 1 (corresponding to the range 60–100% of rhodesain inhibition), an increasingly synergistic action was observed. Moreover, at cellular level we obtained, with the combination synthetic inhibitor + nutraceutical, an antitrypanosomal activity in the low micromolar range and a selectivity index better than that exhibited by **CD24** alone; thus, for all the described reasons, their use in combination is desirable.

## Figures and Tables

**Table 1:** Five selected doses for the combination experiments of **CD24** + curcumin.

Cmps	0.25 × IC_50_	0.5 × IC_50_	IC_50_	2 × IC_50_	4 × IC_50_
CD24	0.05 µM	0.1 µM	0.2 µM	0.4 µM	0.8 µM
Curcumin	3.07 µM	6.15 µM	12.3 µM	24.6 µM	49.2 µM
CD24 + Curcumin	0.05 ± 3.07 µM	0.1 ± 6.15 µM	0.2 ± 12.3 µM	0.4 ± 24.6 µM	0.8 ± 49.2 µM

**Table 2:** Combination index at several f_a_ values.

Fraction Affected (f_a_)	% of Rhodesain Inhibition	Combination Index (*CI*)	Diagnosis of Combined Effect
0.50	50%	1.08	Additive
0.60	60%	0.93	Synergism
0.70	70%	0.81	Synergism
0.80	80%	0.70	Synergism
0.90	90%	0.59	Synergism
1	100%	0.45	Synergism

**Table 3:** Activity against *T. brucei brucei* and HEK293 cells and selectivity index (SI) of **CD24** and curcumin alone and of the combination **CD24** + curcumin.

Compounds	*T. b. brucei* EC_50_ µM	HEK293 EC_50_ µM	SI
**CD24** [29]	10.1 ± 0.5	>70	>6.9
Curcumin [31]	3.12 ± 0.43	>70	>22.4
**CD24** + curcumin	4.85 ± 0.02	>70	>14.4
**Fexinidazole** [38]	2.38 ± 0.88	-	-

## Data Availability

Not applicable.
